# Myocardial Infarction in a Young Female with Palindromic Rheumatism: A Consequence of Negative Remodeling

**DOI:** 10.1155/2012/924141

**Published:** 2012-03-26

**Authors:** Timothy R. Larsen, Sachin Kumar Amruthlal Jain, Jamal Zarghami, Shukri David

**Affiliations:** Department of Internal Medicine, Section of Cardiology Providence Hospital, 16001 West Nine Mile Road, Southfield, MI 48075, USA

## Abstract

Palindromic rheumatism is a rare disease associated with systemic inflammation. Negative or constrictive coronary artery remodeling is typically not seen until the 7th or 8th decade of life. We report a case of a young female with palindromic rheumatism who suffered a non-ST segment elevation myocardial infarction secondary to a flow-limiting lesion that demonstrated negative remodeling by intravascular ultrasound (IVUS).

## 1. Introduction

Palindromic rheumatism is a rare form of inflammatory arthritis characterized by sudden attacks of painful inflammation in one or multiple joints. The attacks last for hours to days and resolve as quickly as they begin [1]. The disease is associated with several markers of systemic inflammation including C-reactive protein, rheumatoid factor, and prolonged erythrocyte sedimentation rate [2]. The chronic inflammatory state associated with this condition can result in unusual presentations of common diseases.

## 2. Case Presentation

A 39-year-old female presented to the emergency room complaining of sudden onset of severe substernal chest pain that started approximately 12 hours prior to presentation. The pain was described as sharp, 9/10 in severity, radiating to the back, associated with diaphoresis and nausea. She admitted to having similar episodes over the previous month that were less severe, which she assumed were “attacks of pleuritis.” These episodes were provoked by exertion and relieved by rest.

Her medical history was significant for palindromic rheumatism diagnosed three years ago; laboratory results from approximately four months prior to presentation showed C-reactive protein of 19 mg/L and erythrocyte sedimentation rate of 40 mm/hour. She was also treated for hypertension; there was no history of diabetes mellitus or dyslipidemia. No prior surgeries. Family history was significant for her mother diagnosed with type II diabetes and gout, her father with coronary artery disease with multiple coronary stents and coronary bypass surgery. She is an active smoker with an 11-pack-year history, rarely drinks alcohol, and does not use recreational drugs. Home medications were etanercept 50 mg subcutaneously weekly, metoprolol 100 mg daily, and prednisone 10 mg daily. She had been taking etanercept for approximately one year.

On physical exam, her blood pressure was 153/92 mmHg, heart rate was 75 beats/min, respiratory rate was 14, and oral temperature was 98.8^o^F. BMI was 30, and there were patchy areas of hypopigmentation of the skin. Chest pain was not reproducible or positional. The remainder of the physical exam was unremarkable.

Laboratory results included white blood cell count 15.8 × 10^9^/L; hemoglobin 13.3 g/dL; blood urea nitrogen 11 mg/dL; serum creatinine 0.6 mg/dL; serum sodium 138 mmol/L; serum potassium 3.8 mmol/L; cardiac troponin 0.14 ng/mL. An initial 12-lead surface EKG showed normal sinus rhythm, normal voltage, and no evidence of ischemia or prior infarct. A follow-up EKG showed normal sinus rhythm with symmetric T wave inversions in leads II, aVF, V1, V2, V3, V4, V5, and V6 consistent with inferolateral ischemia. A chest roentgenogram demonstrated normal cardiac size and normal lungs. With typical symptoms, T wave inversion on EKG, and elevated cardiac biomarkers, the patient was diagnosed with a non-ST segment elevation myocardial infarction.

**Figure 1:**
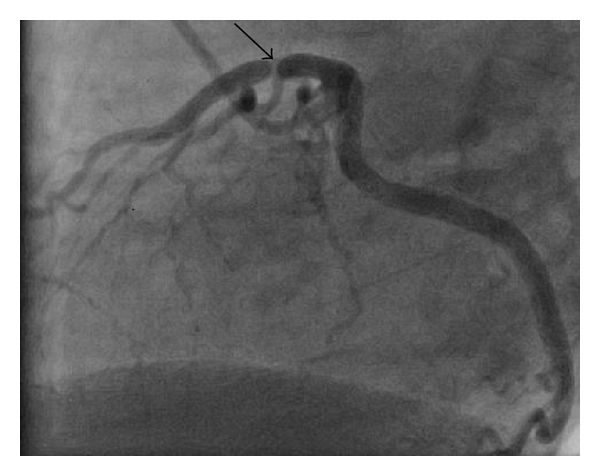
Angiogram of left anterior descending coronary artery demonstrating 80% stenosis of the proximal segment (black arrow).

**Figure 2:**
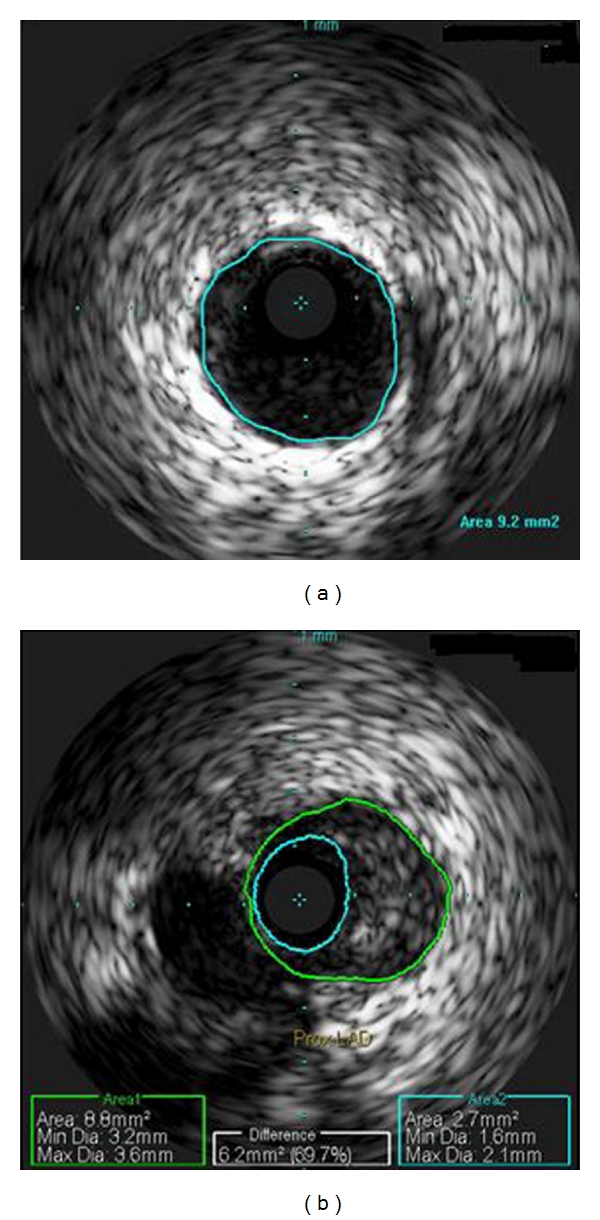
Intravascular ultrasound image of the left anterior descending artery; panel A represents the health proximal reference segment; panel B is the lesion which demonstrates negative remodeling.

The patient was referred for left heart catheterization. Coronary angiogram showed an 80% proximal stenosis of the left anterior descending artery (Figure 1), no disease in the left main, left circumflex, or right coronary arteries, and an ejection fraction of 60%. Intravascular ultrasound revealed evidence of negative remodeling (Figure 2). A bare-metal stent was placed. The patient was observed for 48 hours and discharged home chest-pain-free.

## 3. Discussion

With the use of IVUS the concept of vascular remodeling, first observed in necropsy specimens, has been demonstrated *in vivo*. Positive remodeling is seen when lipid plaques form in the vessel wall resulting in an increased vessel diameter with preserved luminal diameter. Conversely, negative remodeling is a focal narrowing of the vessel lumen [3]. There is a strong association between outward or positive remodeling and plaque rupture leading to acute coronary syndromes including myocardial infarction. Constrictive or negative remodeling appears to be associated with more stable lesions resulting in chronic stable angina [4]. With negative remodeling vessel shrinkage and plaque erosion, rather than plaque rupture with acute thrombosis, are responsible for the ischemia that develops.

Several studies have demonstrated a difference in lesion morphology between the elderly and patients less than 65 years of age. In patients less than 65 years of age, acute coronary syndromes are typically the result of positive remodeled lesions and plaque rupture. Hassani et al. determined positive remodeling to be present in 56% of lesions in younger patients as opposed to 19% of elderly patients (*P* < 0.001) [5]. Calcified plaque and negative remodeling were found to be more common in the elderly. Older age has also been demonstrated as a predictor for negative remodeling [6]. For a 39-year-old female to develop a flow limiting lesion with characteristics similar to what is typically seen in the elderly, there must be an additional process involved.

Negative remodeling results from inflammation within the vessel wall. It has been proposed that the trigger for inflammation is the accumulation of oxidized lipids along with hemodynamic strain [7]. In response, endothelial and intimal cells begin to express adhesion molecules and inflammatory cytokines, which recruit and activate macrophages, monocytes, and lymphocytes. These cells produce additional proinflammatory cytokines which perpetuate the inflammatory cascade. The immune system has powerful regulators that diminish this inflammatory response and therefore are protective against atherosclerosis (mainly interleukin-10 and transforming growth factor *β*). Chronic systemic inflammation tips this balance in favor of atherosclerotic progression. Both erythrocyte sedimentation rate (ESR) and C-reactive protein (CRP) are markers of systemic inflammation. Our patient had an elevated CRP at 19 mg/L approximately four months prior to presentation; 10 mg/L represents the upper 99th percentile in healthy young adult blood donors [8]. Her ESR was 40 mm/hour; normal for a 39-year-old female is less than 25 mm/hour [9]. Thus, this patient had evidence for a significant inflammatory burden, despite therapy with a corticosteroid and tumor necrosis factor-alpha inhibitor. Unfortunately it was not possible to demonstrate focal inflammation at the site of the atherosclerotic lesion, which is recognized as a limitation.

## 4. Conclusion

This young female developed a hemodynamically significant coronary artery lesion which resulted in a non-ST segment elevation myocardial infarction. Likely, the chronic systemic inflammatory state associated with palindromic rheumatism accelerated the process of negative remodeling, which typically takes decades to develop.

## References

[1] Guerne PA, Weisman MH (1992). Palindromic rheumatism: part of or apart from the spectrum of rheumatoid arthritis. *American Journal of Medicine*.

[2] Grassi W, De Angelis R, Lamanna G, Cervini C (1998). The clinical features of rheumatoid arthritis. *European Journal of Radiology*.

[3] Klein LW (2007). Atherosclerosis regression, vascular remodeling, and plaque stabilization. *Journal of the American College of Cardiology*.

[4] Schoenhagen P, Ziada KM, Vince DG, Nissen SE, Tuzcu EM (2001). Arterial remodeling and coronary artery disease: The concept of “Dilated” versus “Obstructive” coronary atherosclerosis. *Journal of the American College of Cardiology*.

[5] Hassani SE, Mintz GS, Fong HS (2006). Negative remodeling and calcified plaque in octogenarians with acute myocardial infarction. An intravascular ultrasound analysis. *Journal of the American College of Cardiology*.

[6] Gyongyosi M, Yang P, Hassan A (1999). Coronary risk factors influence plaque morphology in patients with unstable angina. *Coronary Artery Disease*.

[7] Hansson GK (2005). Mechanisms of disease: Inflammation, atherosclerosis, and coronary artery disease. *The New England Journal of Medicine*.

[8] Pepys MB, Hirschfield GM (2003). C-reactive protein: a critical update. *The Journal of Clinical Investigation*.

[9] Miller A, Green M, Robinson D (1983). Simple rule for calculating normal erythrocyte sedimentation rate. *British Medical Journal*.

